# The incidence and worsening of newly diagnosed low back pain in a population of young male military recruits

**DOI:** 10.1186/s12891-016-1136-2

**Published:** 2016-07-13

**Authors:** Shlomo Moshe, Oren Zack, Aharon S. Finestone, Menashe Mishal, Noa Segal, Dan Slodownik, Yaron Yagev

**Affiliations:** The Occupational Department, Maccabi Healthcare Services, 43 Geulim St, Holon, 5840419 Israel; Department of Environmental and Occupational Medicine, Sackler Faculty of Medicine, School of Public Health, Tel Aviv University, Tel Aviv, Israel; The Israel Defense Forces, Medical Corps, Tel Aviv, Israel; Department of Orthopedics, Assaf Harofeh Medical Center, Zerifin, Affiliated to the Sackler Faculty of Medicine, Tel Aviv University, Ramat Aviv, Israel; Department of Dermatology, Sackler Faculty of Medicine, Sourasky Medical Center, Tel Aviv, Israel; Maccabi Healthcare Services, The Occupational Clinic, Beer-Sheva, Israel; Ben-Gurion University Medical School, Beer Sheva, Israel

**Keywords:** LBP, Incidence, Prevalence, Occupational exposure, Risks, Epidemiology, Young adults, Army recruits, Recrudescence, History

## Abstract

**Background:**

Low back pain (LBP) is a leading cause of referral to occupational health clinics and of consequent work absenteeism. There is lack of data concerning ages 18–21. The objective of our study was to evaluate the occurrence of newly diagnosed LBP and the recurrence and worsening of preexisting LBP in young male military recruits.

**Methods:**

In this retrospective cohort study, we examined the medical history of army recruits during the 30-month period after their induction into the Israel Defense Forces (IDF). The duty status of soldiers in combat units (CU), maintenance units (MU) and administrative units (AU) was evaluated according to their morbidity. The study’s end point was defined as significant findings on clinical examination with presence of neurological deficits which correlate to radiological findings on CT or MRI showing herniated disks, spinal stenosis or pressure on neurological roots.

**Results:**

The annual incidence rate of LBP in a total of 159,295 recruits was 0.05 %. The relative risk (RR) for developing LBP was significantly higher among subjects who were assigned to AU as compared to CU and MU in all LBP categories. The RR for LBP recurrence in soldiers with a positive history of LBP (categories 3 and 4) was 4.1 and 10.7 compare to category 1 respectively.

**Conclusions:**

The lower than expected overall incidence rate of 0.05 % reflects the fact that severe LBP occurrences are not common at this age group. This finding is a more truthful reflection of LBP occurrence rates relative to other studies since the end point is based on precise clinical definitions in medical records and not on questionnaires, as in most studies. The RR for developing LBP was significantly higher among subjects who were assigned to AU as compared to CU and MU in all LBP categories. Childhood history of LBP was found as a significant risk factor for LBP exacerbations at adulthood. Positive history of LBP was found as a risk factor for the recurrence of LBP in all occupation types and particularly in sedentary ones.

## Background

Low back pain (LBP) is one of the most common health problems, creating substantial personal, social, and financial burdens globally [1]. Recent global review of the prevalence of LBP in the adult general population showed a point prevalence rate of 11.9 % with a 1-month prevalence estimated at 23.2 % [2]. In health facility or clinic-based studies, episode remission at 1 year was reported to be between 54 and 90 % [1]. Data from the National Health Interview Survey indicate that there are over 22 million cases of LBP annually in the US that last 1 week or more, resulting in almost 150 million lost workdays [3]. Worldwide, 37 % of LBP is estimated to be attributed to occupational risk factors. The attributable risk fraction is higher for males then for females, largely because of men’s higher participation in the labor force and in occupations with heavy lifting and whole body vibration [3]. Epidemiological studies have reported the prevalence of LBP to be low in children (1–6 %); however, it rises sharply in adolescents (18–51 %) approaching the prevalence in adults [4]. While LBP has been comprehensively researched in adults, it is only more recently that this condition has been studied in children and adolescents [4].

The main biomechanical risk factors identified for the development of LBP at work are heavy physical work, awkward static and dynamic working postures, whole body vibration, and lifting [5, 6]. The psychosocial risk factors identified are negative affectivity, low level of job control, high psychological demands, and high work dissatisfaction [5, 6].

Military service in the Israel Defense Forces (IDF) is mandatory at the age 18 except for those identified as belonging to the subpopulation of Muslims, female orthodox Jews, and all ultra-orthodox Jews. Many individuals enter military service with undiagnosed LBP or mild LBP, which consequently limits performance of their duty [7]. Military service could be challenging to individuals with LBP due to exacerbating factors common in the military environment [7, 8].

In order to understand the contributing factors for the occurrence, and worsening of LBP during military service, we evaluated the impact of various army occupations and different LBP categories over the course of 30 months of compulsory military service in a group of new army recruits aged 18–21.

## Methods

### Data source

This study was planned as a retrospective cohort study. The study population consisted of 18-year-old male Israeli military recruits to the IDF from January 1^st^ 1995 through December 31^st^ 2004. Females were not included in this study since they did not serve in combat units (CU) and not many served in maintenance units (MU). The military recruits were required to attend the IDF’s recruiting office for a medical evaluation. The inductee’s medical history was obtained in most cases from their family physician. During the initial examination, each was asked specifically whether he was ever diagnosed as having LBP problems. If the inductee (or the family physician) gave a positive reply implying the possibility of past or present LBP, he was referred to an evaluation by a certified orthopedic surgeon. The inductee was asked to provide medical information pertinent to the LBP including medications, X-ray or CT imaging studies, EMG, etc. To formalize this process, a board of military physicians then reviewed each recruit’s medical records collected since birth, which includes an examination to establish and approve a functional limitation grade classification. The follow-up was limited to 30 months.

### Medical categories

The subjects were divided into the following categories according to criteria written in the IDF's book of medical profiles: 1 - All subjects that have no evidence of past and/or present LBP; 2 - Mild scoliosis or kyphosis, Negative medical history for LBP; 3-Positive medical history for LBP, no clinical findings, normal X-rays; 4 - Positive medical history for LBP, no clinical findings, X-ray with mild changes; 5 -Positive medical history for LBP, clinical findings with neurological deficit, CT or MRI showing herniated disks or spinal stenosis or pressure on neurological roots; 6 – Severe back pain with neurological deficit or severe functional limitation but good prognosis for improvement; 7 - Severe back pain with a significant neurological deficit and severe functional limitation. Subjects in Categories 6–7 were not drafted to the IDF. To clarify, Categories 1 and 2 represent common findings without any pain complaints. Categories 3 and 4 represents positive LBP history, i.e., the data represents development of a new episode of LBP for Categories 1 or 2, or the worsening of a previous condition of LBP for Categories 3 or 4.

### Occupational categories

The soldiers’ assignments were categorized into CU, MU, and administrative units (AU). These categories were created by the army’s manpower system in order achieve the best correlation between the inductee’s limitations and efforts needed in each category. The CU service included infantry CU, with strenuous physical activity and high mental stress, and mobile CU with less strenuous physical activity and shorter training durations. The MU service is characterized by a more moderate physical activity and moderate mental stress (mechanics, welders, etc.) The AU service is predominantly characterized by sedentary office work and low mental stress. We used these categories as markers of occupational stress.

Soldiers in medical Categories 1 and 2 were posted in all military units. Soldiers in Category 3 were posted in CU (in less demanding tasks), MU or AU. Soldiers in Categories 4 were posted in either MU or AU only and those in category 5 were assigned to AU only. There are almost no rotations in job assignment during service as long as the soldiers are fit for their duty.

### Follow up during the service

All subjects posted in either CU, MU or AU units were followed for 30 months by the units’ physicians noting newly diagnosed LBP. If any symptoms suggestive of LBP developed during the follow-up period, the subjects were reevaluated by a certified orthopedic surgeon in accordance with the medical parameters defined in the military medical book of profiles. The medical status was then reassessed by a military medical committee comprised of two senior physicians. If necessary, the medical profile was adjusted according to the new evidence and the soldier was reassigned to a suitable post. The study endpoint per subject was defined as a change in the medical profile from the preliminary Categories 1–4 to either category 5–7, i.e. the development of a new episode of LBP for Category 1 or worsening of previous condition of LBP (Categories 2–4). Almost all inductees with significant medical condition were categorized in Category 5. This process was supervised and monitored by a trained physician stationed at the headquarters of the IDF medical corps.

### Data analysis

The relative risks (RR) and Fisher’s 95 % confidence intervals for new onset LBP during the study period with subsequent drop in medical profile were estimated by comparing incidence rates using stratified analysis according to disease categories. A *p* < 0.05 in two tailed tests was considered to be significant. All analyses were conducted using a standard statistical package (Compare 2 version 2.97, Copyright JH Abramson 2000–2001).

## Results

The survey included 204,866 18-year-old recruits divided into 4 categories. The total number of inductees in Categories 1–4 were 159,295, 35,281, 6,701 and 3,589 respectively. The average age of the cohort was 19.06 ± 1.4 years. Table 1 summarizes the annual incidence rates of LBP during the study period according to the subject’s status at recruitment. The annual incidence for newly diagnosed LBP (category 1, Table 1) was 0.05 %, 0.046 % and 0.08 % in CU, MU, and AU respectively. The RR for developing LBP was significantly higher among subjects in Category 1 who were assigned to AU compared to CU and MU (RR = 1.64 and 1.81, respectively *p* < 0.05) (Table 1).

**Table 1:** Relative risk (RR) and 95 % confidence interval (CI) for new-onset or recurrent LBP among young male soldiers (18–21 years), by severity of disease at recruitment for the follow up period and per 1 year

Severity of LBP	Military service	Incident cases (IR^a^) for 1 years	RR (95 % CI)	*P*
Category 1 - Low Back Pain rates in a healthy population of new recruits	AU (5,914)	49 (0.083 %)	1.6 (1.2–2.1)	<.001
	MU (52,330)	240 (0.046 %)	0.9 (0.9–1.3)	NS
	CU (101,051)	510 (0.050 %)	1	
Total category 1	159,295	799 (0.050 %)		
Category 2 - LBP rates in a population with minor clinical findings	AU (1,350)	26 (1.93 %)	2.6 (1.6–3.6)	<.001
	MU (12,470)	64 (0.51 %)	0.7 (0.5–0.9)	<.001
	CU (22,001)	165 (0.75 %)	1	NS
Total category 2	35281	255 (0.71 %)		
Category 3 - LBP rates in a population with positive medical history and no significant radiology findings	AU (457)	22 (4.81 %)	2.4 (1.4–3.5)	<.001
	MU (3,213)	54 (1.68 %)	0.9 (0.6–1.25)	NS
	CU (3,031)	60 (1.98 %)	1	
Total category 3	6701	136 (2.03 %)		
Category 4 - LBP rates in a population with positive medical history and minimal radiologic findings	AU (381)	43 (11.29 %)	2.2 (1.5–2.8)	<.001
	MU (1,749)	74 (4.23 %)	0.8 (0.6–1.1)	NS
	CU (1,459)	76 (5.21 %)	1	
Total category 4	3589	193 (5.38 %)		

*CU* combat units, *MU* maintenance units, *AU* administrative units, *NS* non significant

^a^Crude incidence rate (%) of new or recurrent cases of LBP during military service

The annual incidence for LBP recurrence among soldiers who had mild scoliosis or kyphosis with negative medical history for LBP (Category 2, Table 1) was 0.75 %, 0.51 % and 1.93 % in CU, MU, and AU respectively. The RR for developing LBP was significantly higher among subjects who were assigned to AU as compared to CU and MU (RR = 2.57 and 3.75 respectively, *p* < 0.05).

The annual incidence of LBP diagnosis in soldiers who had a positive medical history and minimal radiologic findings (Category 3, Table 1) was 5.2 %, 4.2 % and 11.3 % in CU, MU, and AU respectively. The RR for developing LBP was significantly higher among subjects who were assigned to AU as compared to CU and MU (RR = 2.2 and 2.7 respectively *p* < 0.05).

The RR for development of severe LBP during the follow up period is shown in Table 2. The RR comparing soldiers in Categories 2 to 1 was 1.4 (95 % CI = 1.2–1.6), in categories 3 to 1 was 4.1 (95 % CI = 3.4–4.7), and in Categories 4 to 1 was 10.7 (95 % CI = 9.3–12.2).

**Table 2:** The RR and Confidence Interval of developing or worsening new-onset or recurrence of LBP within the same types of military duty in varying medical profiles^a^

Category	Combat unit	Maintenance unit	Administrative unit	Total
2/1	1.4 (1.2–1.6)	1.1 (0.8–1.4)	2.3 (1.4–3.3)	1.4 (1.2–1.6)
3/1	4.1 (3.8–4.7)	3.7 (2.7–4.6)	5.8 (3.4–8.2)	4.1 (3.4–4.7)
4/1	10.7 (9.3–12.2)	9.2 (7.2–11.3)	13.6 (9.8–17.5)	10.7 (9.3–12.2)
3/2	2.6 (2.0–3.3)	3.3 (2.4–4.2)	2.5 (1.4–3.6)	2.9 (2.4–3.5)
4/2	7.0 (5.4–8.5)	8.3 (6.4–10.1)	5.9 (4.2–7.6)	7.6 (6.5–8.6)
4/3	2.6 (2.0–3.3)	2.5 (1.9–3.1)	2.3 (2.0–3.1)	2.7 (2.3–3.1)

1- All subjects that have no evidence of past and/or present LBP

2 - Mild scoliosis or kyphosis; negative medical history for LBP

3- Positive medical history for LBP; no clinical findings; normal X-rays

4- Positive medical history for LBP; no clinical findings; X-ray with mild changes

^a^The RR is calculated by dividing incidence rates. For example the rate between category 1 to category 0 (1/0) is calculated by dividing the incidence in category 1 (0.99 %) to the incidence in category 0 (0.041 %); the result is 24.8

## Discussion

In this retrospective cohort study, we evaluated 204,866 military conscripts representing more than 50 % of a given age group of healthy Israeli males at age 18, over a 2.5-year follow-up period between the years 1995–2004. The annual incidence of newly diagnosed LBP in Category 1 individuals was 0.05 %. The RR for developing LBP was significantly higher among subjects who were assigned to AU as compared to CU and MU in all LBP categories. The RR for recurrence of LBP comparing Category 3 (population with positive history for LBP) to 1 (healthy population of new recruits) was 4.1. These figures clearly demonstrate the role of a positive medical history of LBP as a predictor for recrudescence of the disease.

The annual incidence of LBP in the general population of the US is about 1–5 % [1–3]. Knox et al. [8] found an overall incidence rate (IR) for LBP of 40.5 per 1000-person-years (i.e. IR ~ 4 %) in the US army. A higher rate was found among enlisted service members (4.8 %) probably because they are typically involved in more physically demanding occupations and physical training regimens that place significant strain on the lower back. Mattila et al. [9] investigated the incidence and trends of LBP hospitalization among Finnish military conscripts. Hospital admission rates due to unspecified LBP was 19.1 per 1,000-person-years (i.e. ~ 2 %) and 7.8 per 1,000 person-years (0.8 %) due to lumbar disc disorders. Waterman et al. [10] showed that the incidence of acute LBP requiring medical evaluation in the emergency department is 1.39 per 1,000-person-years (i.e. ~ 0.14 %). In a study by Ernat et al. [11], the unadjusted rate of LBP for enlisted infantrymen was 35.2 per 1,000 person-years (3.52 %). Most prevalence studies on LBP are based on self-answered questionnaires or mailed surveys with no objective criteria. Subjects answering questionnaires may tend to exaggerate their complaints [12, 13]. On the other hand, medical boards responsible for drafting recruits might underestimate the severity of the back pain complaints and ensuing limitations on function [7]. The difference between these studies and our study is the end point, defined herein as a change in medical profile, which translates to the presence of neurological deficits and radiological (CT or MRI) evidence showing either herniated disks, spinal stenosis or pressure on neurological roots (Category 5); conversely, in other studies the end point is the first occasion in which the diagnosis was written in the medical records [8, 11], hospitalizations [9, 10] or incidence reported by questionnaires [12, 13]. Other factors which could contribute to the low incidence of LBP are unwillingness to report in a military environment, difficulty of reporting to supervisors, physician’s disinclination to disqualify, etc. In this study we were not able to estimate the impact of these factors; however, judging from our experience in the army, we assume these factors to have a minimal effect. To summarize this point, the different end point (more stringent than other studies), the young population age and the fact that this study relies on medical records and not questionnaires explains why the incidence rates in our study (0.05 %) are closer to the reported incidence of severe LBP rates (0.14–3.52 %) and lower than expected overall LBP rates.

The RR for severe LBP comparing Categories 3 and 4 to category 1 was 4.1 and 10.7 respectively (Table 2). The RR for severe LBP is higher with a previous history of LBP and even higher when minimal radiologic findings are found. Inferred from our current data are two questions: a. whether LBP in childhood and adolescence predisposes to LBP in adulthood, b. what is the role of a positive medical history as a predictor for LBP in new employees and particularly in those working in strenuous physical activities. Although very few longitudinal studies exist, Harreby et al. [14] showed that schoolchildren reporting LBP in their growth period and familial occurrence of back disease are important risk factors for LBP later in life, with an observed probability of 88 % if both factors are present. In a 13-year follow-up study of 335 children aged 8–17 years, reporting of back pain at recruitment did not predict back pain at follow up, but did predict the reporting of pain (generally) in adulthood [15]. Studies of other pain syndromes in childhood, such as abdominal pain or headache, on which there is more data, show consistently that there is a future risk of the specific pain syndrome, medically unexplained syndromes, or adverse psychological outcomes [16]. It seems that our results are coherent with the above literature.

There is strong evidence that the single, most consistent, predictor of further LBP and work loss in pre-placement assessment is a previous history of LBP [17, 18]. Himelstein et al. [17] reviewed in the late eighties the role of pre-placement screening in LBP. They found that there was a significant increased risk of LBP after many episodes of LBP, many sickness absence days because of LBP short intervals between episodes or after an aggravated course of LBP. Thus, information not only about the occurrence of previous LBP but also about the severity has predictive value. Videman et al. [19] investigated the prevalence of LBP from entering nursing school through 5 years among 174 nursing students and found an association with LBP history at the entry to nursing school (OR = 7.1). The results from our study showing a higher risk for severe LBP with previous history of LBP, corroborate this finding.

The RR for developing LBP was significantly higher among subjects who were assigned to AU as compared to CU and MU in all medical categories (Table 2), even though AU are mostly involved in sedentary work while MU and CU are engaged with more strenuous activities. Hartvigsen et al. [20], in a review including 35 original articles, evaluated the roll of sitting as a risk for LBP and concluded that all of the studies but one failed to find a positive association between sitting-while-working and LBP. In contrast, Van Nieuwenhuyse et al. [21] evaluated risk factors for first-ever LBP among workers in their first employment and found that an increased risk was observed for long periods of seated work (RR = 3.2). Studies which examined military populations found different results. Ernat et al. [11] found that the unadjusted rate for LBP at enlisted infantrymen was 35.2 per 1,000 person-years (3.52 %), higher than AU (0.083 %). Lincoln et al. [22] examined the data of 15,268 active-duty personnel and found that lower pay grade, musculoskeletal diagnosis, shorter length of service, older age, occupational category, lower job satisfaction, recurrent musculoskeletal hospitalizations, greater work stress, and heavier physical demands are risk factors of musculoskeletal conditions resulting in disability among US army personnel. Feuerstein et al. [23] found similar results. Carragee et al. [12] demonstrated that many soldiers feel that LBP is a normal part of their occupation and therefore tend to ignore or minimize this condition. To summarize, the reason for higher RR rates of LBP in AU is unclear but likely multifactorial. One likely possibility is a decreased willingness to seek treatment in the probably more motivated CU population. The expectations of meeting certain professional and physical demands as well as the idea of succumbing to injury, are psychosocial factors that this subgroup may face, preventing them from seeking a medical provider. Another potential factor is the training and fitness level required of the CU soldier, which may provide a protective effect against the significant loads and forces placed on their lower backs. However, this remains unclear and represents avenues for further research in this population.

The present study has several strengths. The first being that it is based on large figures (about 200,000 conscripts) which is an order of magnitude (1–2 times higher) than most other studies in this field. Second, the definition of LBP is clear and is based on a decision made by a medical military committee. Third, LBP data were collected from computerized data, guaranteeing a high coverage of LBP because all inductees were recorded in a computerized system. Fourth, the design of the study is a historical prospective follow-up of 2.5 years. Fifth, all soldiers served in obligatory service, i.e. they were in low ranks. Finally, the military environment provided highly standardized conditions for investigating the effects of occupational risk factors: conscripts trained in the same area, ate the same food and lived in the same barracks with nearly equal daily military programs for each occupation. There are some drawbacks in this study. There is lack of data concerning psychosocial parameters, weight, and smoking habits, which can influence LBP as shown in other studies. The study includes only male recruits and that is why we can draw conclusions only about males.

## Conclusions

The present study was comprised of 204,866 military conscripts, representing about 50–70 % of a given age group of healthy Israeli males, and provides insights for a better understanding of the factors underlying the development of a first episode of LBP in adulthood. When looking at the severe LBP cases, we found an incidence rate of only 0.05 % among the study population. This is a clearly lower than expected rate. We believe this finding to be a more truthful reflection of LBP occurrence rates relative to other studies since the end point is based on precise clinical definitions in medical records and not on questionnaires, as in most studies [12, 13]. The strongest risk factor found for LBP was the history of LBP, a renowned fact [17–20]. The lower incidence rate of LBP in the CU population may reflect a higher motivation and better fitness in this subgroup. The higher incidence rate of LBP in AU is surprising and requires further research.

## Abbreviations

AU, administrative units; CU, combat units; IDF, Israel Defense Forces; LBP, low back pain; MU, maintenance units; RR, relative risk
